# On-chip photonic Fourier transform with surface plasmon polaritons

**DOI:** 10.1038/lsa.2016.34

**Published:** 2016-02-26

**Authors:** Shan Shan Kou, Guanghui Yuan, Qian Wang, Luping Du, Eugeniu Balaur, Daohua Zhang, Dingyuan Tang, Brian Abbey, Xiao-Cong Yuan, Jiao Lin

**Affiliations:** 1 Department of Chemistry and Physics, La Trobe Institute for Molecular Science (LIMS), La Trobe University, Melbourne, VIC 3086, Australia; 2 Australian Research Council Centre of Excellence for Advanced Molecular Imaging, Australia; 3 School of Physics, The University of Melbourne, VIC 3010, Australia; 4 Centre for Disruptive Photonic Technologies, Nanyang Technological University, Singapore 637371, Singapore; 5 Institute of Materials Research and Engineering, A*STAR, 3 Research Link, Singapore 117602, Singapore; 6 Nanophotonics Research Centre, Shenzhen University & Key Laboratory of Optoelectronic Devices and Systems of Ministry of Education and Guangdong Province, College of Optoelectronic Engineering, Shenzhen University, Shenzhen 518060, China; 7 School of Electrical and Electronic Engineering, Nanyang Technological University, Singapore 639798, Singapore; 8 School of Engineering, RMIT University, Melbourne, VIC 3001, Australia

**Keywords:** diffraction, Fourier optics, optical computing, optical information processing, surface plasmon polaritons

## Abstract

The Fourier transform (FT), a cornerstone of optical processing, enables rapid evaluation of fundamental mathematical operations, such as derivatives and integrals. Conventionally, a converging lens performs an optical FT in free space when light passes through it. The speed of the transformation is limited by the thickness and the focal length of the lens. By using the wave nature of surface plasmon polaritons (SPPs), here we demonstrate that the FT can be implemented in a planar configuration with a minimal propagation distance of around 10 μm, resulting in an increase of speed by four to five orders of magnitude. The photonic FT was tested by synthesizing intricate SPP waves with their Fourier components. The reduced dimensionality in the minuscule device allows the future development of an ultrafast on-chip photonic information processing platform for large-scale optical computing.

## Introduction

*Fourier analysis* is the process of decomposing a general function into the sum of a set of simpler periodic functions, which corresponds to the mathematical operation of a Fourier transform (FT). It gives rise to many fundamental principles in physics, engineering, and mathematics^1^. For instance, spectroscopic measurement of the *spectrum* (FT of the waveform in the time domain) of white light shows the energy distribution over the constituent electromagnetic waves of different frequencies. As for a monochromatic electromagnetic waveform, the FT of the spatial distribution of the field gives the *angular spectrum*, where each point in the spectrum represents a constituent planewave propagating in a specific direction^2,3^. On the other hand, *Fourier synthesis*, the process opposite to Fourier analysis, reconstructs a function from its spectrum using the inverse FT (IFT). Differentiated only by a parity operator, the IFT and FT can, in practice, be implemented using the same physical process. In optics, it is remarkably simple that a single converging lens performs the complicated FT/IFT at the speed of light, such that the complex amplitudes in the front and back focal planes become an FT pair (with some additional scaling factors) ^4^. This lays the foundation for most experimental works in the area of Fourier optics^5,6^, enabling the analysis of the angular spectrum of light and the synthesis of light in the reverse process. The optical FT carried out by a lens also forms a basis of optical computing^7,8^ due to its parallelism and unrivalled speed, which is ultimately limited by the size of the optical system. Recently, planar structures have been shown to be able to perform mathematical operations as light passes through, providing a possibility to replace conventional lens-based optical systems, but the fundamental principle is still based on free-space optics^9^. In fact, the physical footprint of such optical information processing systems could be reduced substantially if these operations could be carried out solely in a two-dimensional (2D) space rather than in free-space. Plasmonic nanostructures offer a rare opportunity to manipulate light at the deep subwavelength scale, which has been demonstrated as a route to substantially reduce the size of many photonic devices^10,11,12,13,14^ including basic optics, such as waveplates^15,16,17^, phase plates^18,19^, and color filters^20,21,22^. Here, we report that the complex operation of an FT can be performed in a single 2D plane via surface plasmon polaritons (SPPs)—a propagating surface wave strongly confined at a dielectric-metal interface, which travel at a velocity very close to the speed of light. Besides the reduced dimensionality, the SPP-based device is able to increase the speed of the photonic FT by several orders of magnitude with a minimal spatial separation between the input and output.

## Materials and methods

### Diffraction integral of SPPs and Fourier relationship

**Figure 1:**
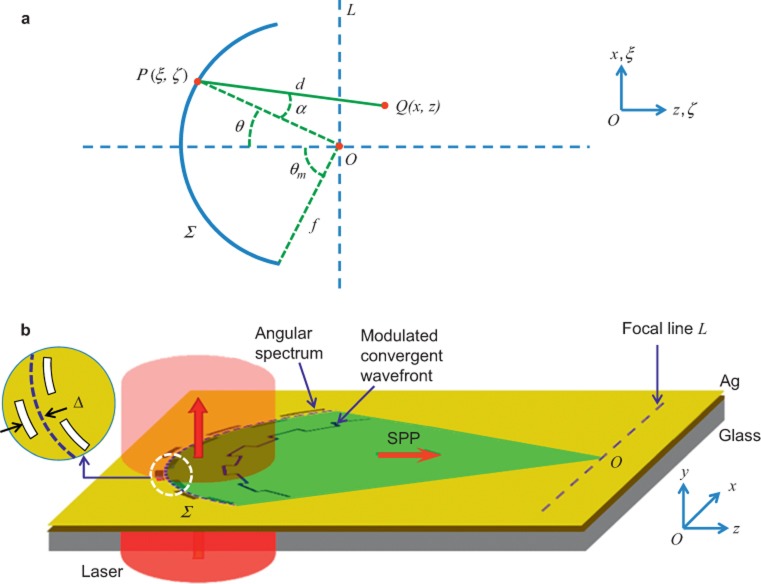
(**a**) Schematics of in-plane FT performed by surface wave propagation. In a plane, the disturbance at any point *Q*(*x,z*) in the vicinity of focus *O*(0,0) can be calculated by summing up the contributions from all the points, e.g., *P*(*ξ,ζ*) on a convergent wavefront (depicted as the arc Σ with the radius *f*). *d* is the distance between *P* and *Q*. *α* measures the inclination angle of the distance with respect to the normal of the arc. The focal line *L* is perpendicular to the horizontal axis (*z* or *ζ*) across the focus. *θ* is the polar angle in the corresponding polar coordinates. (**b**) Slits in the angular spectrum represented by a reference arc are perforated in the optically opaque metal film (e.g., Ag) on a glass substrate. When illuminated by a coherent light source such as a laser, the slits generate converging SPPs at the air–Ag interface. The converging wavefront is modulated by the displacement Δ(*θ*) of the slits from the reference arc (blue dashed curve).

For monochromatic propagating SPPs at a source-free metal/dielectric interface in the *xz* plane, the out-of-plane electric field *E_y_* (in the dielectric medium) satisfies the 2D Helmholtz equation (the section *Two-dimensional wave equation*, Supplementary Information):

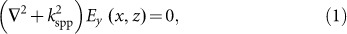


where *k*_spp_ is the wavevector of the SPPs. The FT performed in free space by a converging lens is, in fact, enabled by the spherical wavefront produced by the lens. Despite the change of dimensionality, a parallel relationship can exist for surface waves such as SPPs. Using the 2D form of the integral theorem of Helmholtz and Kirchhoff^23^, we calculate the disturbance at point *Q* in the focal region of a converging cylindrical surface wave as illustrated in Figure 1a provided the focal length (>10 μm) is much larger than the wavelength of the SPPs (the section *Propagation integral for SPPs*, Supplementary Information):

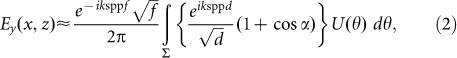


where *U*(*θ*) is the complex amplitude on the arc Σ of a reference circle with radius *f*, the distance between *P*(*ξ, ζ*) on Σ and *Q*(*x, z*) is 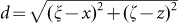, and *α* is the inclination angle of the distance with respect to the normal of the arc. We discover that, to a good approximation, a 2D Fourier relationship can be found for the complex amplitude *U*(*θ*) along an arc of a converging surface wave and the field distribution *E_y_*(*x, z*) in the vicinity of the geometrical focus (the section *Two-dimensional Fourier relationship for a converging SPP*, Supplementary Information):

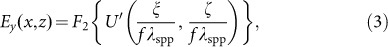


with 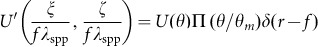, where *λ*_spp_ is the wavelength of the SPP; 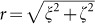; *θ_m_* is the maximal half polar angle of the arc; Π(•) and δ(•) are the rectangular function and the Dirac delta function, respectively; *F_n_*{•} denotes the *n*-dimensional FT.

### Projection-slice theorem of SPPs in the focal plane

Consequently, using the projection-slice theorem, we find a one-dimensional (1D) Fourier relationship between the field distribution on the focal line *L* (perpendicular to the propagation axis) and the projection of the complex amplitude *U*(*θ*) onto the same line *L* (the section *Projection-slice theorem*, Supplementary Information):

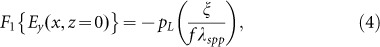


where 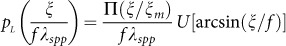 is the projection of 
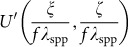
 onto the focal line *L* and this expression is valid near the focus.

This result is instrumental to understanding the present work and we use it here to demonstrate the following examples of Fourier synthesis of a surface wave *E*_y_(*x, z*) from its angular spectrum. This is made possible through controlling the input *U*(*θ*) on a reference arc, since a monochromatic directional surface wave can be fully represented by its transverse profile *E_y_*(*x, z* = 0).

In Figure 1b, we implement *U*(*θ*) on an arc consisting of discrete subwavelength slits equally spaced in the azimuthal direction. When illuminated by a laser beam, the ensemble of slits produces SPPs which form a convergent wavefront. Each slit is displaced in the radial direction from the reference arc by 
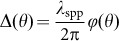
, where *ϕ(θ)* is the phase of *U*(*θ*). To simplify the design, we use phase-only functions for *U*(*θ*), since the spectral phase alone is sufficient to synthesize most of surface waves with finite transverse dimensions^24^. The spectral amplitude can be modulated in the design by controlling the width of slits.

### Sample preparation and fabrication

After depositing a 300 nm thick Ag layer onto a silica substrate via electron beam evaporation, we milled the subwavelength slits with a nominal width of 240 nm through the Ag film using a focused ion beam (Zeiss, AURIGA 60). The Ag film is thick enough to entirely block the incident beam, since the penetration depth of 632.8 nm light is just 24 nm in Ag (*ε_Ag_* = – 15.93 + 1.077*i*). This helps to improve the signal-to-noise ratio of the measured near-field intensity distribution of SPPs. The radius of the reference arc is 15 µm which is a balance between the number of slits and the propagation loss (the SPP propagation length at an Ag/air interface is around 22 µm at this wavelength). In the experiment, these subwavelength slits are designed in a discrete pattern composed of several segments.

### Near-field intensity distribution measurements

The intensity distributions of SPP waves were measured by a near-field scanning optical microscope (NSOM, NT-MDT/NTEGRIS Solaris) equipped with an aluminum-coated fiber tip with a 100 nm diameter aperture working in collection mode. The laser was first expanded by a telescope system to a ∼10 mm diameter spot before slightly focused by a low numerical aperture objective lens (4×, NA = 0.13) onto the sample surface, incident from the substrate side. SPPs are excited when the slits are illuminated with the laser beam. Detailed experimental setup is given in the section *Experimental setup for measurement* of the Supplementary Information.

## Results and discussion

### Synthesis of plasmonic Weber beam in the Fourier space

First we synthesized a new type of SPP wave dubbed the plasmonic Weber beam (PWB), which we prove to be a rigorous non-diffracting solution of the 1D Helmholtz equation in parabolic cylindrical coordinates (the section *Derivation of plasmonic Weber beams in parabolic cylindrical coordinates*, Supplementary Information), giving an explicit form of the dominant electric field *E_y_* at *z* = 0 as:

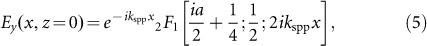


where 2*k*_spp_*a* is the defined separation constant, and _2_*F*_1_[*a*; *b*; *v*] is the confluent hypergeometric function of the first kind. The complex angular spectrum of the PWB is given by (sections *Derivation of plasmonic Weber beams in parabolic cylindrical coordinates* and *Angular spectrum of the PWB*, Supplementary Information):

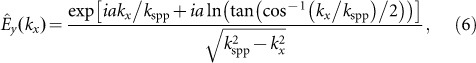


where *k_x_* = *k*_spp_ sin *θ* is the transverse wavevector. Thus the corresponding offset of the slits (Figure 1b) from the reference arc is given by:

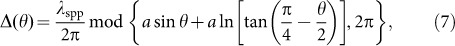


where mod{*p*, *q*} gives the remainder after division of *p* by *q*.

### Near-field measurement of PWB

In the experiment, we used a linearly polarized He-Ne laser with wavelength of 632.8 nm as the coherent source to excite SPPs. Subsequently, these converging SPP waves propagate and interfere at the geometrical focus forming the PWB, and its near-field intensity distribution is measured by NSOM. We choose an angular spectrum ranging from −60° to 60° for the Fourier synthesis of the PWB. The effect of the truncated spectrum on the reconstruction is minimal as the main feature of the PWB is preserved (the section *Dependence of PWB on angular range of nanostructure*, Supplementary Information).

**Figure 2:**
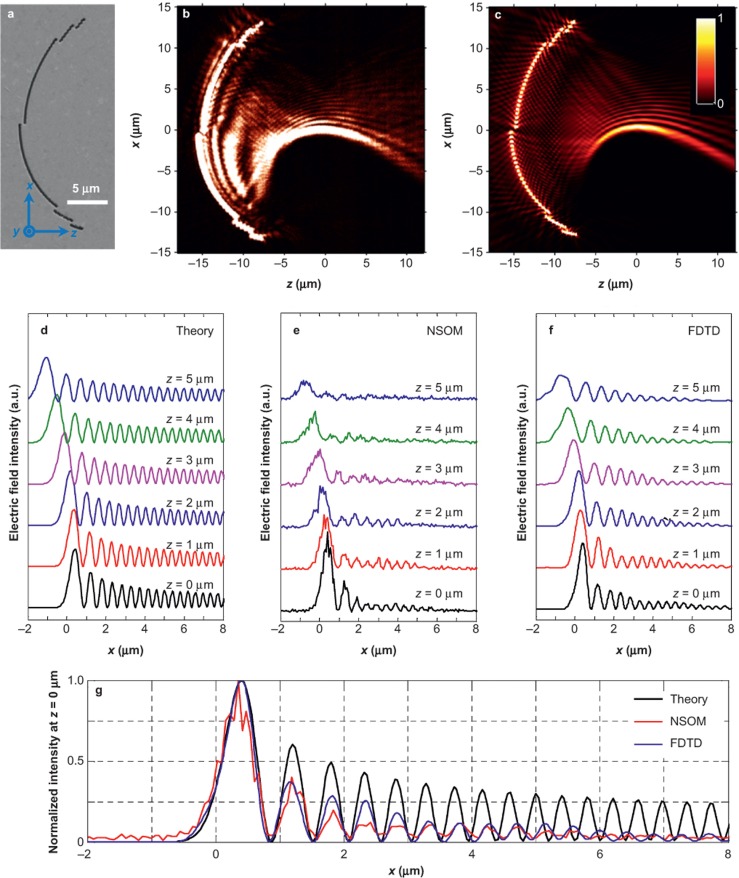
(**a**) SEM micrograph of the plasmonic nanostructure in the angular spectrum of a PWB (*a* = 40), consisting of slits (240 nm in width) perforated into a 300 nm thick silver film and fabricated by FIB (Zeiss, 30 kV). The radial spatial shift with respect to reference arc (radius of 15 μm) determines the relative phase in the angular spectrum. The incident beam (*λ* = 632.8 nm) is *z*-polarized and illuminates the nanostructure from the substrate side. (**b)** NSOM measurement and (**c**) FDTD calculations results of electric field intensity distributions, showing excellent agreement in-between. The snapshots of the beam profiles at various propagation distances from 0 to 5 µm in a step of 1 µm obtained from theory, NSOM, and FDTD are shown in (**d**), (**e**), and (**f**), respectively. (**g**) Comparison of the normalized intensity distribution in the focal line (*z* = 0) in the cases of theory, NSOM, and FDTD.

Figure 2a gives the scanning electron microscope micrograph of the first plasmonic nanostructure with *a* = 40. The NSOM measurement results and supporting numerical calculations using the finite-difference time-domain (FDTD) method in Figure 2b and 2c are in good agreement. The synthetic PWBs propagate along curved trajectories for more than 10 µm beyond the focal line. To verify that the SPP wave is indeed a PWB, we studied its evolution dynamics along its propagation direction and make quantitative comparisons to the experimental results, FDTD simulations, and the theoretical predictions (the section *Derivation of plasmonic Weber beams in parabolic cylindrical coordinates*, Supplementary Information for the explicit expression of the PWB). The snapshots of the electric field intensity distributions along the +*z* direction ranging from 0 to 5 µm in steps of 1 µm are juxtaposed in Figure 2d–2f accordingly. All the profiles exhibit the self-bending property consistent with the theory. For example, from *z=*0 to *z* = 4 μm, the lateral shifts of the main peak from NSOM, FDTD, and theory are measured as 1.1, 1.16 and 1.48 µm, respectively. However, the intensity of the side lobes in the NSOM and FDTD decreases much faster than the corresponding theoretical prediction. This is attributed to the additional amplitude modulation induced by the polarization selectivity of the slits (the section *Angular spectrum of the PWB*, Supplementary Information). The arc radius also has a small influence on the properties of the generated PWBs. Due to the spatial extension of the PWB, the actual angular spectrum at points far away from the geometrical center will deviate from the theoretical prediction given by Equation (6). This causes the PWB’s trajectory to be laterally shifted from the original design (the section *Effect of the arc radius on the PWB properties*, Supplementary Information). In order to improve the excitation efficiency of the SPPs, the structures can be repeated. For example, three periods with separation of *λ*_spp_ can improve the excitation efficiency by six times and the signal-to-noise ratio is much better (the section *PWB generation at different geometric parameters (single-period slit of a = 60 and multiple-period slits*), Supplementary Information).

### Synthesis and experimental characterization of plasmonic Airy beam

**Figure 3:**
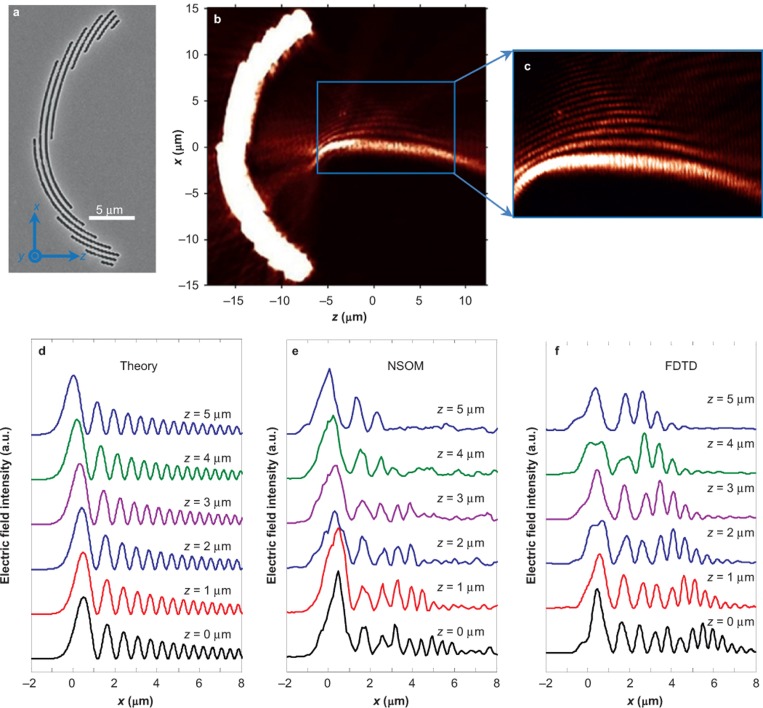
(**a**) SEM micrograph of three-period nanostructures to synthesize the PAB, which is a special case of the PWB under paraxial approximation. *x*_0_=0.5 μm, *r*_0_=15 μm, and *b*=0.02. (**b**) NSOM measurement results of near-field electric field intensity distribution. (**c**) Zoom-in view of the blue box area in (**b**). For quantitative comparison and comprehensive evaluation of the beam properties, we give the line-to-line intensity distributions from 0 to 5 µm in a step of 1 µm obtained from the analytical formula, NSOM measurement, and FDTD calculation in (**d**), (**e**), and (**f**), respectively.

Besides the synthesis of a new surface wave from its angular spectrum, the proposed on-chip FT also simplifies the generation of some well-known surface waves, especially when the angular spectrum of the desired wave is simple. A good example is the Plasmonic Airy Beam (PAB)^25,26,27^ whose angular spectrum can be represented as^28^:

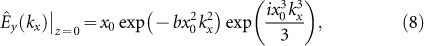


where the parameter *b* ≪ 1 describes the exponential apodization of the field profile and *x*_0_ determines the width of the main lobe. The angular spectrum is essentially a Gaussian distribution modified by a cubic phase term. Therefore, using the same Fourier synthesis procedure we can implement the specific cubic phase in the angular spectrum for the generation of a PAB. Taking the parameters as *x*_0_ = 0.5 μm and *b* = 0.02, we end up with the design shown in Figure 3a, where a three-period design was utilized to provide a better coupling efficiency. At the same polar angle, slits from various reference arcs of different radii are in-phase and thus will provide the same Fourier components in the direction of the focus. The experimental near-field intensity distributions are shown in Figure 3b. The Airy waveform is observed close to the focal plane with the characteristic non-diffracting and self-bending features. Overall consistency has been observed in Figure 3d–3f despite slight discrepancies that can be attributed to the contributions from the slits at large angles beyond the paraxial approximation implied by PABs.

## Conclusions

In summary, we have established a Fourier relationship embedded in the propagation of surface waves, which enables a photonic FT/IFT performed with SPPs. This allows one to design FT-based planar devices to be incorporated into existing integrated optics, marking another step toward on-chip optical computing. Compared with the optical FT in free space, the on-chip configuration plasmonic nanostructures offer four to five orders of magnitude of enhanced processing speed due to the reduced footprint of the device. Considering the short focal length (∼10 μm) of a converging surface wave, the FT can now be finished in tens of femtoseconds. Besides the promising applications in optical computing, one can further synthesize intricate surface waves and even create new exotic surface waveforms (e.g., designer SPPs) to accommodate the wide range of applications of SPPs^29,30,31,32^.

## Authors’ contributions

S.S.K., G.Y., and J.L. designed the experiments, interpreted the results, and prepared the manuscript. E.B. and B.A. prepared the samples. Q.W., L.D., D.Z., D.T., and X.Y. carried out the experiments. All authors commented on the manuscript.

## Competing financial interests

The authors declare no competing financial interests.
