# Supplementary material for: On-chip photonic Fourier transform with surface plasmon polaritons
Source: Light Sci Appl. 2016 Feb 26;5(2):e16034–. doi: 10.1038/lsa.2016.34 (PMC6062422; doi:10.1038/lsa.2016.34)
Supplement: Supplementary information [file lsa201634x1.pdf]

Supplementary Information for

On-chip photonic Fourier transform with surface plasmon polaritons

Shan Shan Kou,† Guanghui Yuan,† Qian Wang, Luping Du, Eugeniu Balaur, Daohua Zhang, Dingyuan Tang, Brian Abbey, Xiao-Cong Yuan*, Jiao Lin*

†These authors contributed equally to this work.

*Correspondence to: xcyuan@szu.edu.cn or jiao.lin@osamember.org

Section A: Two-dimensional wave equation

Assuming the surface plasmon polaritons (SPPs) propagate at a planar interface ($y=0$) between a dielectric of permittivity ε_d and a metallic substrate of permittivity ε_m, the longitudinal component of electric field E_y should obey the scalar Helmholtz equation:

$$\nabla^2 E_y + k_0^2 \varepsilon_d E_y = 0 \tag{S1}$$

where $k_0 = 2\pi/\lambda$ is the wave vector in free space and the time dependence term $e^{-i\omega t}$ is neglected. Since the electric field of SPPs exponentially decays in the perpendicular direction with respect to the interface, we assume that the electric field has the general form:

$$E_y(x,y,z) = A(x,z)e^{i(k_x\cdot x + k_z\cdot z)}e^{-\alpha_d\cdot y} \tag{S2}$$

where the in-plane wavevector $k_{spp} = \sqrt{k_x^2 + k_z^2} = k_0\sqrt{\frac{\varepsilon_m\varepsilon_d}{\varepsilon_m + \varepsilon_d}}$ and $\alpha_d = \sqrt{k_{spp}^2 - k_0^2\varepsilon_d} = k_0\sqrt{\frac{\varepsilon_d^2}{-(\varepsilon_m + \varepsilon_d)}}$.

By considering $\frac{\partial^2 E_y}{\partial y^2} = \alpha_d^2 E_y$, we have

$$\frac{\partial^2 E_y}{\partial x^2} + \frac{\partial^2 E_y}{\partial z^2} + k_{spp}^2 E_y = 0 \tag{S3}$$

Section B: Propagation integral for SPPs

Using the Weber's analogue of the Helmholtz-Kirchhoff theorem[1], the disturbance at *Q* (Fig. S1) for a convergent SPP can be given by

$$E_y(x,z) = -\frac{i}{4}\int_{S=\Sigma+\Omega_1+\Omega_2+\Gamma}\left\{\upsilon\frac{\partial H_0^{(1)}(k_{spp}d)}{\partial n} - H_0^{(1)}(k_{spp}d)\frac{\partial\upsilon}{\partial n}\right\}ds\,, \qquad \text{(S4)}$$

where υ is the field distribution on the closed curve consisting of Σ, Ω_1, Ω_2 and Γ; *d* is the distance between *P* and *Q*; $H_m^{(1)}(x)$ is the *m*th order Hankel function of the first kind; $\frac{\partial}{\partial n}$ means the differentiation along the outward normal to the closed curve. If we have a truncated converging wave on the arc Σ, the field distribution on Ω_1 and Ω_2 approximately satisfies $\upsilon = 0$ and $\frac{\partial\upsilon}{\partial n} = 0$. On the other hand, since the surface wave travels at a finite speed, we will assume that the field distribution on Γ is zero at the time of measurement as the radius $R \to \infty$. Therefore, the disturbance at *Q* results from the single contribution from the arc Σ:

$$E_y(x,z) = -\frac{i}{4}\int_{\Sigma}\left\{\upsilon\frac{\partial H_0^{(1)}(k_{spp}d)}{\partial n} - H_0^{(1)}(k_{spp}d)\frac{\partial\upsilon}{\partial n}\right\}ds\,. \qquad \text{(S5)}$$

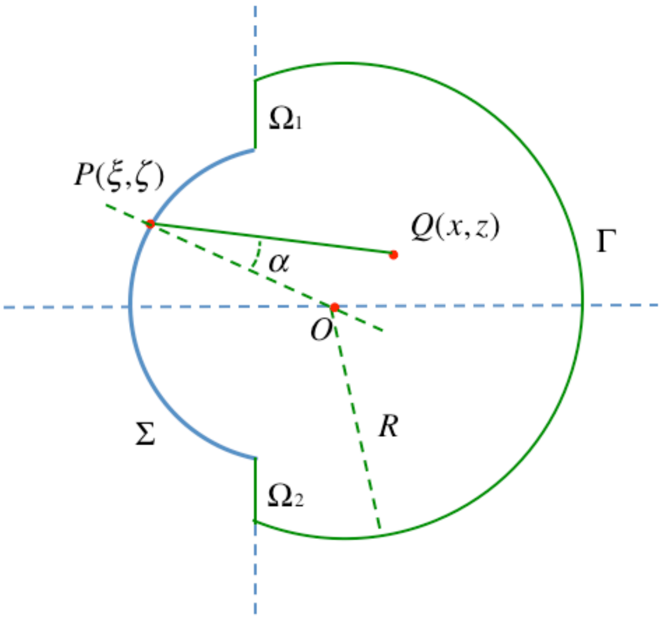

Figure S1: Derivation of the propagation integral of surface waves. The closed curve consists of the reference arc Σ representing the convergent wavefront, an arc Γ of a large circle of radius *R*, and two portions Ω_1 and Ω_2 that connect the two arcs.

Assuming a general converging cylindrical wave $\upsilon(r,\theta)=U(\theta)H_0^{(2)}(kr)$ in polar coordinates (r,θ), where $H_m^{(2)}(x)$ is the *m*th order Hankel function of the second kind, we obtain:

$$E_y(x,z)=i\frac{k_{spp}f}{4}\int_\Sigma\left\{H_0^{(2)}(k_{spp}f)H_1^{(1)}(k_{spp}d)\cos\alpha-H_1^{(2)}(k_{spp}f)H_0^{(1)}(k_{spp}d)\right\}U(\theta)\,d\theta\,. \qquad \text{(S6)}$$

Here, the following relationships are used: $ds=fd\theta$, $\frac{d}{dx}H_0^{(1)}(x)=-H_1^{(1)}(x)$ and $\frac{d}{dx}H_0^{(2)}(x)=-H_1^{(2)}(x)$.

If f ? λ_{spp} and d ? λ_{spp}, Eq. (S6) can be simplified:

$$E_y(x,z)\approx\frac{e^{-ik_{spp}f}\sqrt{f}}{2\pi}\int_\Sigma\left\{\frac{e^{ik_{spp}d}}{\sqrt{d}}(1+\cos\alpha)\right\}U(\theta)\,d\theta \qquad \text{(S7)}$$

by using the asymptotic forms of the Hankel functions[2]: $H_n^{(1)}(x)\sim\sqrt{\frac{2}{\pi x}}e^{i\left(x-\frac{n}{2}\pi-\frac{\pi}{4}\right)}$ and $H_n^{(2)}(x)\sim\sqrt{\frac{2}{\pi x}}e^{-i\left(x-\frac{n}{2}\pi-\frac{\pi}{4}\right)}$.

<u>**Section C**: Two-dimensional Fourier relationship for a converging SPP</u>

For the field distribution near the focus, $\alpha\approx 0$. Thus, we have

$$E_y(x,z)\approx\frac{e^{-ik_{spp}f}\sqrt{f}}{\pi}\int_\Sigma U(\theta)\frac{e^{ik_{spp}d}}{\sqrt{d}}\,d\theta\,, \qquad \text{(S8)}$$

which can be regarded as a 2D integral:

$$E_y(x,z)=\int_{-\pi}^{\pi}\int_0^{\infty}U(\theta)\Pi(\theta/\theta_m)\delta(r-f)\frac{e^{ik_{spp}d}}{\sqrt{d}}rdrd\theta \qquad \text{(S9)}$$

where θ_m is the maximal half polar angle of the arc and $\Pi(\cdot)$ and $\delta(\cdot)$ are the rectangular function and the Dirac delta function, respectively. Using the following approximation:

$$d=\sqrt{(\xi-x)^2+(\zeta-z)^2}=\sqrt{f^2+x^2+z^2-2(\xi x+\zeta z)}\approx f\sqrt{1-\frac{2}{f^2}(\xi x+\zeta z)}$$

$$\approx f\left[1-\frac{1}{f^2}\left(\xi x+\zeta z\right)\right]=f-\frac{\xi x+\zeta z}{f} \tag{S10}$$

and neglecting the imaginary part of k_{spp} since it mainly contributes to the propagation loss that affects the focal field strength relatively uniformly from all directions, we have

$$E_y\left(x,z\right)=\int_{-\pi}^{\pi}\int_{0}^{\infty}U\left(\theta\right)\Pi\left(\theta/\theta_m\right)\delta\left(r-f\right)\exp\left[-i\frac{k_{spp}}{f}\left(\xi x+\zeta z\right)\right]rdrd\theta$$

$$\approx\int_{-\infty}^{\infty}\int_{-\infty}^{\infty}U'\left(\xi',\zeta'\right)\exp\left[-i2\pi\left(\xi' x+\zeta' z\right)\right]d\xi' d\zeta' \tag{S11}$$

where $U'\left(\xi',\zeta'\right)=U\left(\theta\right)\Pi\left(\theta/\theta_m\right)\delta\left(r-f\right)$; $\xi'=\frac{\mathrm{Re}\left(k_{spp}\right)}{2\pi f}\xi=\frac{1}{f\lambda_{spp}}\xi$,

$\zeta'=\frac{\mathrm{Re}\left(k_{spp}\right)}{2\pi f}\zeta=\frac{1}{f\lambda_{spp}}\zeta$.

Neglecting the constant factor gives:

$$E_y\left(x,z\right)=\mathcal{F}_2\left\{U'(\xi',\zeta')\right\} \tag{S12}$$

Section D: Projection-slice theorem

The electric field distribution on the focal line *L* shown in Fig. 1b can be calculated using the projection-slice theorem.

$$p\left(\xi'\right)=\int_{-\infty}^{\infty}U'\left(\xi',\zeta'\right)d\zeta'=\int_{-\infty}^{\infty}U\left(\theta\right)\Pi\left(\theta/\theta_m\right)\delta\left(r-f\right)d\zeta'$$

$$=\frac{1}{f\lambda_{spp}}U\left[\arcsin\left(\lambda_{spp}\xi'\right)\right]\Pi\left[\frac{\arcsin\left(\lambda_{spp}\xi'\right)}{\theta_m}\right]$$

$$=\frac{1}{f\lambda_{spp}}U\left[\arcsin\left(\xi/f\right)\right]\Pi\left[\xi/\xi_m\right] \tag{S13}$$

where $\xi_m=2f\sin\left(\theta_m/2\right)$ and $\xi=f\lambda_{spp}\xi'$. Therefore from (S11), we have

$$E_y\left(x,z=0\right)\approx\int_{-\infty}^{\infty}\int_{-\infty}^{\infty}U'\left(\xi',\zeta'\right)\exp\left[-i2\pi\xi' x\right]d\xi' d\zeta'$$

$$= \int_{-\infty}^{\infty} \left\{ \int_{-\infty}^{\infty} U'\left(\xi', \zeta'\right) d\zeta' \right\} \exp\left[-i2\pi\xi' x\right] d\xi'$$

$$= \int_{-\infty}^{\infty} p\left(\xi'\right) \exp\left[-i2\pi\xi' x\right] d\xi'$$

Substituting (S13) into above integral and expressing it as 1D Fourier transform, we have

$$E_y\left(x; z=0\right) = \frac{1}{f\lambda_{spp}} \mathcal{F}_1\left\{ U\left[\arcsin\left(\lambda_{spp}\xi'\right)\right] \Pi\left[f\lambda_{spp}\xi' / \xi_m\right] \right\} \quad \text{(S14)}$$

namely,

$$E_y(x; z=0) = \mathcal{F}_1\left\{ p_L\left(\frac{\xi}{f\lambda_{spp}}\right) \right\}, \quad \text{(S15)}$$

where $p_L\left(\frac{\xi}{f\lambda_{spp}}\right) = \frac{\Pi\left(\xi/\xi_m\right)}{f\lambda_{spp}} U\left[\arcsin(\xi/f)\right]$ is the projection of $U'\left(\frac{\xi}{f\lambda_{spp}}, \frac{\zeta}{f\lambda_{spp}}\right)$ onto the focal line L and $\xi' = \frac{\xi}{f\lambda_{spp}}$. Applying the FT to Eq.(S15), we will have

$$\mathcal{F}_1\left\{E_y(x; z=0)\right\} = -p_L\left(\frac{\xi}{f\lambda_{spp}}\right). \quad \text{(S16)}$$

Section E: Experimental setup for measurement

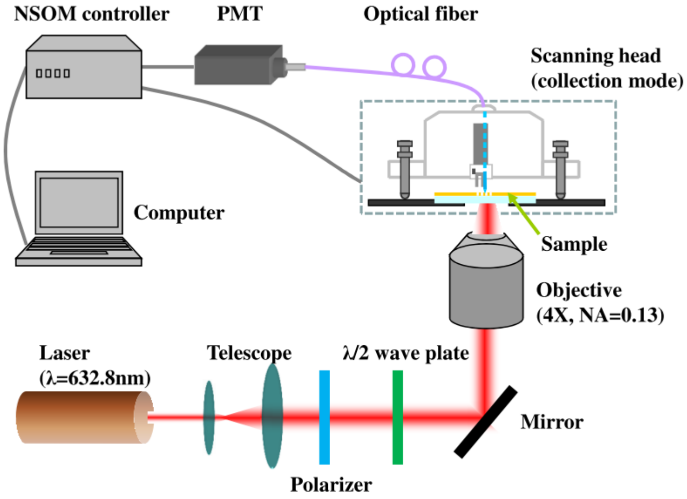

Figure S2: Experimental setup for near-field measurement.

The experimental setup for registering the SPP fields at the air/Ag interface is shown in Figure S2. A linearly polarized laser beam with wavelength of λ=632.8nm was expanded and collimated by a telescope system, and subsequently passes through a linear polarizer and a half wave plate to get the desired linear polarization state. In order to improve the signal intensity, the laser beam is slightly focused by an objective (Olympus UPlanFLN 10×, NA=0.3) and illuminates onto the sample from the substrate side. A near-field scanning optical microscope (NSOM, NT-MDT NTEGRA Solaris) is used to image the SPP fields on top surface of the samples by raster scanning an aluminum-coated fiber probe with a 100 nm-diameter aperture. A non-optical shear-force feedback mechanism is used to perform the distance control between the sample and fiber tip, typically less than 20 nm. Sequentially, the near-field evanescent SPP field is sampled by the probe and converted into guided modes within an optical fiber, the other end of which is connected to a photo-multiplier tube (PMT) for signal detection and amplification.

Section F: Derivation of plasmonic Weber beams in parabolic cylindrical coordinates

Equation (S3) is a two-dimensional equation that is separable in Cartesian, circular cylindrical, elliptical cylindrical and parabolic cylindrical coordinates[3,4]. In parabolic cylindrical coordinates (σ,τ,y) are defined by $x+iz=(\sigma+i\tau)^2/2$ and $y=y$ with ranges $\sigma\in(-\infty,\infty);\tau\in[0,\infty);y\in(0,\infty)$. Equation (S3) then becomes

$$\frac{\partial^2 E_y}{\partial\sigma^2}+\frac{\partial^2 E_y}{\partial\tau^2}+(\sigma^2+\tau^2)k_{spp}^2E_y=0 \tag{S17}$$

which has a factored solution $E_y=F(\sigma)G(\tau)e^{-\alpha_d\cdot y}$. After substituting it into (S17), we can get

$$\frac{1}{F(\sigma)}\left[\frac{\partial^2F(\sigma)}{\partial\sigma^2}+\sigma^2k_{spp}^2F(\sigma)\right]+\frac{1}{G(\tau)}\left[\frac{\partial^2G(\tau)}{\partial\tau^2}+\tau^2k_{spp}^2G(\tau)\right]=0$$

Since these two terms are functions of independent variables σ and τ, they can be separated by defining a separation constant as

$$\frac{1}{F(\sigma)}\left[\frac{\partial^2F(\sigma)}{\partial\sigma^2}+\sigma^2k_{spp}^2F(\sigma)\right]=-2k_{spp}a \text{ and } \frac{1}{G(\tau)}\left[\frac{\partial^2G(\tau)}{\partial\tau^2}+\tau^2k_{spp}^2G(\tau)\right]=2k_{spp}a$$

with the transverse part $F(\sigma)$ and $G(\tau)$ satisfying the following differential equations:

$$\frac{\partial^2 F(\sigma)}{\partial \sigma^2}+\left(\sigma^2 k_{spp}^2+2k_{spp}a\right)F(\sigma)=0 \tag{S18a}$$

$$\frac{\partial^2 G(\tau)}{\partial \tau^2}+\left(\tau^2 k_{spp}^2-2k_{spp}a\right)G(\tau)=0 \tag{S18b}$$

where $2k_{spp}a$ is the separation constant. Equations (S18a) and (S18b) are similar as the equations (7a) and (7b) in Ref. 5 which demonstrate the three-dimensional propagation-invariant Weber accelerating beams (WABs) in parabolic cylindrical coordinate in free-space. In our case, $k_{spp}=(1.033+0.002i)k_0$ with the imaginary part mainly affecting the resultant field strength. Therefore, by neglecting the contribution of the imaginary contribution to the Fourier transform, the angular spectrum of the plasmonic Weber beam (PWB) can, in a similar way, be expressed as:

$$\hat{E}(k_x)|_{z=0}=\frac{exp\left[iak_x/k_{spp}+ia\ln\left(\tan\left(\cos^{-1}\left(k_x/k_{spp}\right)/2\right)\right)\right]}{\sqrt{k_{spp}^2-k_x^2}} \tag{S19}$$

The explicit form of PWB is derived in the following. The even and odd solutions of the differential equation $\frac{d^2P}{dv^2}-\left(\frac{v^2}{4}+a\right)P=0$ are given by[3]:

$$even: P_e(v,a)=e^{-\frac{v^2}{4}}{}_2F_1\left[\frac{a}{2}+\frac{1}{4};\frac{1}{2};\frac{v^2}{2}\right] \tag{S20a}$$

$$odd: P_o(v,a)=ve^{-\frac{v^2}{4}}{}_2F_1\left[\frac{a}{2}+\frac{3}{4};\frac{3}{2};\frac{v^2}{2}\right] \tag{S20b}$$

where ${}_2F_1[a;b;v]$ is the confluent hypergeometric function of the first kind.

Using the coordinate transformation $v\rightarrow ve^{\frac{i\pi}{4}}, a\rightarrow ia$, the solutions of the differential equation $\frac{d^2P}{dv^2}+\left(\frac{v^2}{4}+a\right)P=0$ can be expressed as:

$$even: P_e^{'}(v,a)=e^{-\frac{iv^2}{4}}{}_2F_1\left[\frac{ia}{2}+\frac{1}{4};\frac{1}{2};\frac{iv^2}{2}\right] \tag{S21a}$$

$$odd: P_o^{'}(v,a)=ve^{-\frac{iv^2}{4}}{}_2F_1\left[\frac{ia}{2}+\frac{3}{4};\frac{3}{2};\frac{iv^2}{2}\right] \tag{S21b}$$

Using the coordinate transformation $v=\sqrt{2k_{spp}}\sigma$, equation (S18a) becomes

$$\frac{d^2F(v)}{dv^2}+\left(\frac{v^2}{4}+a\right)F(v)=0 \tag{S22}$$

The even and odd solutions are given by:

$$F_e(\sigma)=e^{-\frac{iv^2}{4}}{}_2F_1\left[\frac{ia}{2}+\frac{1}{4};\frac{1}{2};\frac{iv^2}{2}\right]=e^{-\frac{ik_{spp}\sigma^2}{2}}{}_2F_1\left[\frac{ia}{2}+\frac{1}{4};\frac{1}{2};ik_{spp}\sigma^2\right] \tag{S23a}$$

$$F_o(\sigma)=\sqrt{2k_{spp}}\sigma e^{-\frac{ik_{spp}\sigma^2}{2}}{}_2F_1\left[\frac{ia}{2}+\frac{3}{4};\frac{3}{2};ik_{spp}\sigma^2\right] \tag{S23b}$$

Similarly, the even and odd solutions of equation (S18b) read as

$$G_e(\tau)=e^{-\frac{ik_{spp}\tau^2}{2}}{}_2F_1\left[-\frac{ia}{2}+\frac{1}{4};\frac{1}{2};ik_{spp}\tau^2\right] \tag{S24a}$$

$$G_o(\tau)=\sqrt{2k_{spp}}\tau e^{-\frac{ik_{spp}\tau^2}{2}}{}_2F_1\left[-\frac{ia}{2}+\frac{3}{4};\frac{3}{2};ik_{spp}\tau^2\right] \tag{S24b}$$

When transferring back to Cartesian coordinates (x,z), using the relationship $z=\sigma\tau$ and $x=(\sigma^2-\tau^2)/2$, the amplitude of the PWB takes the following form at $z=0$

$$E_y(x,y,z=0)=F_e\left(\sqrt{2x}\right)G_e(0)e^{-\alpha_d\cdot y}=e^{-ik_{spp}x}{}_2F_1\left[\frac{ia}{2}+\frac{1}{4};\frac{1}{2};2ik_{spp}x\right]e^{-\alpha_d\cdot y} \tag{S25}$$

The general form of the travelling solution is given by

$$\begin{aligned}E_y(x,y,z)&=\frac{1}{\sqrt{2\pi}}\left[|\Gamma_1|^2F_e(\sigma)G_e(\tau)+2i|\Gamma_3|^2F_o(\sigma)G_o(\tau)\right]e^{-\alpha_d\cdot y}\\&=\frac{1}{\sqrt{2\pi}}e^{-\frac{ik_{spp}(\sigma^2+\tau^2)}{2}}e^{-\alpha_d\cdot y}\left\{|\Gamma_1|^2\times{}_2F_1\left[\frac{ia}{2}+\frac{1}{4};\frac{1}{2};ik_{spp}\sigma^2\right]\times{}_2F_1\left[-\frac{ia}{2}+\frac{1}{4};\frac{1}{2};ik_{spp}\tau^2\right]+\right.\\&\left.2i|\Gamma_3|^2k_{spp}\sigma\tau\times{}_2F_1\left[\frac{ia}{2}+\frac{3}{4};\frac{3}{2};ik_{spp}\sigma^2\right]\times{}_2F_1\left[-\frac{ia}{2}+\frac{3}{4};\frac{3}{2};ik_{spp}\tau^2\right]\right\}\end{aligned} \tag{S26}$$

where $\Gamma_1=\Gamma\left[\frac{ia}{2}+\frac{1}{4}\right]$, $\Gamma_3=\Gamma\left[\frac{ia}{2}+\frac{3}{4}\right]$ and $\Gamma[x]$ is the complex Gamma function.

Section G: Angular spectrum of the PWB

The complex angular spectrum of the PWB is plotted in Fig. S3. Since the spectral phase is highly oscillatory at large polar angles, we only take the central angle range from -60° to 60° arcs indicated by the red line in Fig. S3a, which was found to be sufficient to reproduce the profile of the PWB. Moreover, under our experimental conditions, the actual amplitude of SPPs emanating from the slits is modulated by $\cos\theta$ as shown in Fig. S3b due to the polarization selection rules (only the radial in-plane electric field component of the incident beam is transverse-magnetic polarized with respect to the orientation of slits). This additional amplitude modulation modifies the local strength of the transverse profile of a synthesized PWB, i.e. the energy distribution in the mainlobe and sidelobes, but its characteristics in terms of the positions of local maxima and minima are still preserved.

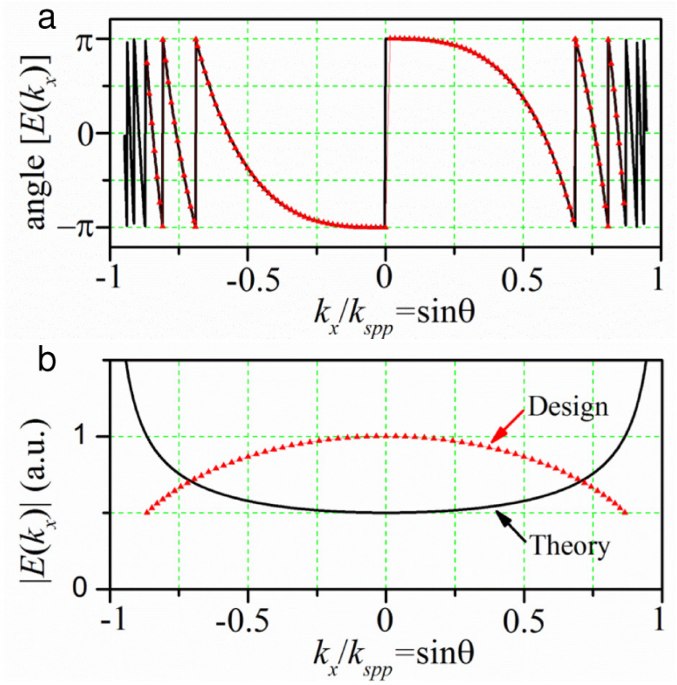

Figure S3: (**a**) Phase and (**b**) amplitude of the angular spectrum of plasmonic Weber beam (PWB) using Fourier synthesis method, where $a = 40$, $r_0 = 15\mu m$ and $\lambda = 632.8nm$. The black solid lines give the theoretical value from Eq. (S19), and the red lines with triangles denote the angular spectrum range used in the experimental design. Note that the phase distribution is exactly the same as the theoretical prediction within the range of $k_x / k_{spp} \in [-0.866, 0.866]$.

We investigated the PWB profiles at the focal plane ($z=0$) for different incident beam sizes and polarization states by use of Eq. (S8), where each slit is viewed as a series of secondary sources emitting cylindrical waves with an initial amplitude dependent on the polarization state and incident beam size.

The calculation results are given in Fig. S4a. For linearly polarized and radially polarized beams and an illumination with a uniform amplitude at the nanostructure (concentric alignment between the beam and arc-center, see inset figure (I) in Fig. S4a), the first five peak positions are almost overlapped with theoretical values. The difference lies in the relative intensity of the mainlobe and sidelobes.

For a Gaussian beam illumination with an electric field modulated by e^{-r^2/w^2} and centrally aligned with the mirror symmetry center of the nanostructure (see inset figure (II) in Fig. S4a), the generated PWB profiles are quite similar to theoretical prediction when $w \geq 20\mu m$. For $w=10\mu m$ and even less, the peaks are slightly shifted and deviate from the theory since the amplitude modulation becomes predominant. Thus we can infer that the additional amplitude modulation induced by the linearly polarized excitation beam with a finite beam size used in the experiment is adequate for retrieving the original PWB.

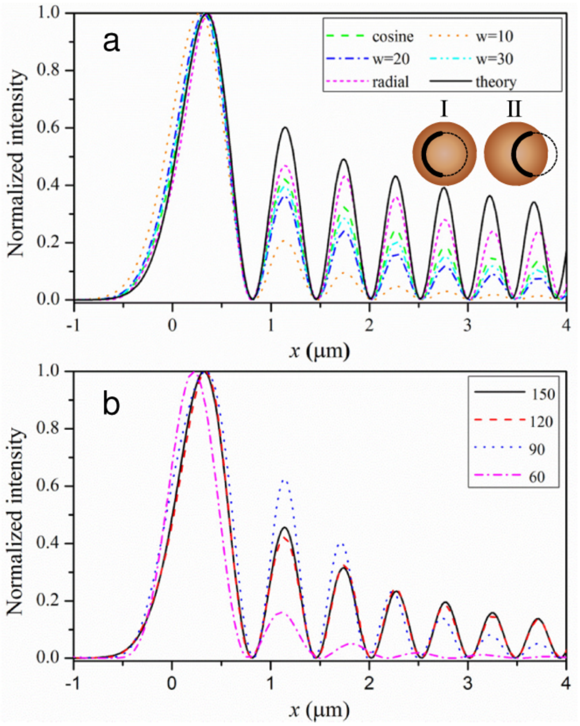

Figure S4: (**a**) The dependence of the amplitude modulation of incident beam on the generated PWBs and comparison with the theoretical results: linear polarization has a cosine-like modulation due to the polarization projection from the *z*-polarization onto the radial component since only the radial electric field component is transverse-magnetic polarized and can excite SPPs; radial polarization has uniform amplitude; the effect of beam size with different radius is shown as well. Inset is shown two general illumination schemes: (I) concentric alignment with uniform amplitude; (II) central alignment of the beam and symmetric centering of the nanostructure with a Gaussian function for the beam amplitude. (**b**) The dependence of the phase modulation of the plasmonic nanostructure with various angular ranges on the generated PWBs.

Section H: Dependence of PWB on angular range of nanostructure

The phase modulation determined by the angular range of the nanostructure will not degrade the generated PWB profiles for an angular range larger than 90°. It can be seen from Fig. S4b that the PWB profiles almost remain the same in the cases of 90°, 120° and 150° angle ranges. The beam properties deviate from the theoretical prediction with a decrease of the angular range. For instance at an angular range of 60°, we observe inconsistency in the peak positions compared to the theory and a rapid reduction in the strength of sidelobes. Therefore, the 120° nanostructure in the experiment provides the sufficient phase modulation required by the PWB.

Section I: Effect of the arc radius on the PWB properties

In the above discussion, the arc radius was chosen to be $r_0 = 15\mu m$. The resulting beam profile of the generated PWB is apparently asymmetric (Fig. 2b) with respect to the Fourier plane. However in the ideal case, the PWB should be symmetric. This discrepancy originates from the fact that the angular spectrum given by Eq. (S19) is only provided near the geometric center of the fabricated nanostructure. Due to the spatial extent of the PWB, the actual angular spectrum far away from the center will deviate from the theoretical value (different θ and transverse wavevector), resulting in the degradation of the predicted PWB. This deviation can be compensated by use of a larger arc radius. We have calculated the electric field intensity distributions at larger r_0, whilst keeping other structural parameters such as a, slit width and the angular range remain unchanged. The calculated results are shown in Figs. S5a-5e.

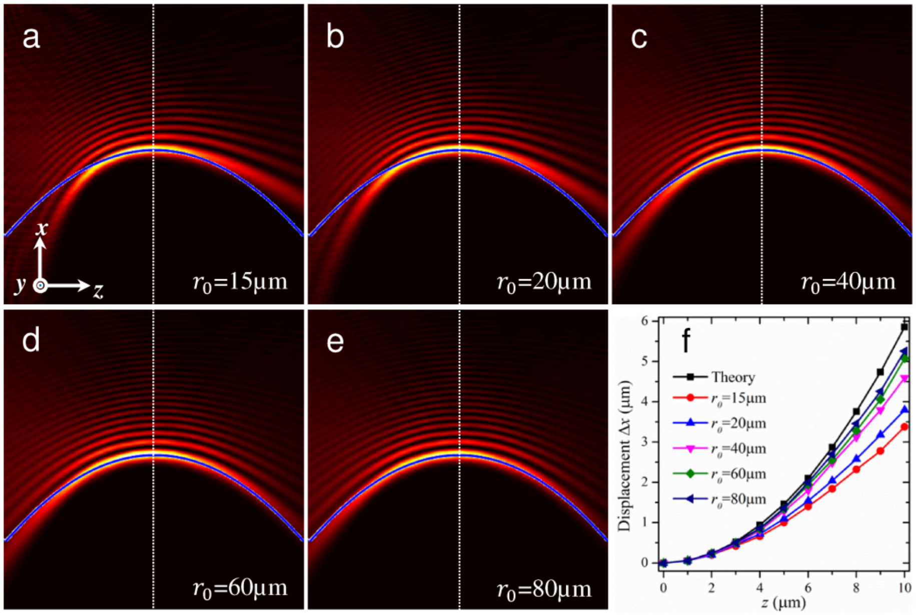

Figure S5: PWB profile for different arc radii r_0: (**a**) 15μm, (**b**) 20μm, (**c**) 40μm, (**d**) 60μm and (**e**) 80μm, where the vertical dotted lines show the Fourier plane and the solid blue lines indicate the theoretical trajectory of the main peak predicted by equation (S26). The quantitative comparison of the results of the displacement of the main peak as a function of propagation distance is depicted in (**f**).

Guided by the electric field intensity data, we studied the lateral displacement of the main peak as a function of the propagation distance for different r_0 and made quantitative comparisons with the theory. The data are presented in Fig. S5f. With each increment of r_0, the beam trajectory increasingly approaches the values given by the theoretical curves. By fitting the displacement with a parabolic function $\Delta x = -\beta \times z^2$, the constant β is evaluated to 0.035μm^{-1}, 0.04μm^{-1}, 0.048μm^{-1}, 0.051μm^{-1} and 0.053μm^{-1} in the cases of r_0 at 15μm, 20μm, 40μm, 60μm and 80μm respectively. In comparison, the theoretical constant β is 0.058μm^{-1}.

It is also worth noting that, after the focal plane, the beam width increases monotonically in the direction of propagation, indicating the diverging nature of the PWB in Cartesian coordinates. This behaviour can be understood easily since this beam is nondiffracting only in the parabolic cylindrical coordinate system.

Section J: PWB generation at different geometric parameters (single-period slit of *a*=60 and multiple-period slits)

We utilized a similar Fourier synthesis method to the one described above to generate the PWB using other geometrical parameters. The results for a single-period slit of $a = 60$ are depicted in Fig. S6. Both the NSOM measurement and FDTD calculations show reasonable agreement with theory, providing similar trajectories and overall beam profiles. There are some intensity fluctuations in the NSOM results however, which are due to the point-by-point scanning by the finite aperture size of the tip. These artifacts can be significantly reduced by increasing the period of the slits. For example, three-period-slit nanostructures improve the excitation efficiency of the PWB to be about 6 times higher than that of single-period slit, providing a much better signal-to-noise ratio of the electric field intensity profile as shown in Fig. S7.

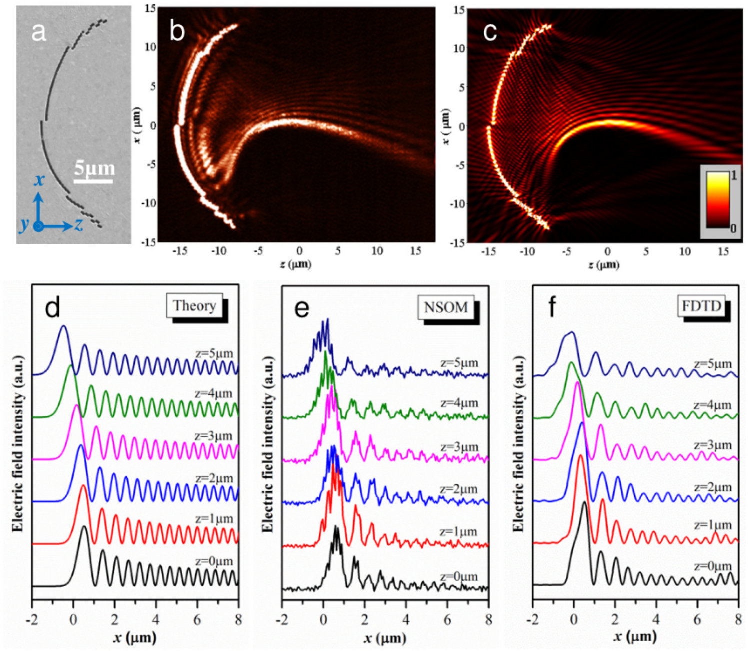

Figure S6: (**a**) SEM micrograph of single-period nanostructures used to synthesize the PWB for $a = 60$. (**b**) NSOM measurement and (**c**) FDTD calculation results of electric field intensity distributions. The snapshots of the beam profiles at various propagation distances obtained from theory, NSOM and FDTD are shown in (**d**), (**e**) and (**f**) respectively.

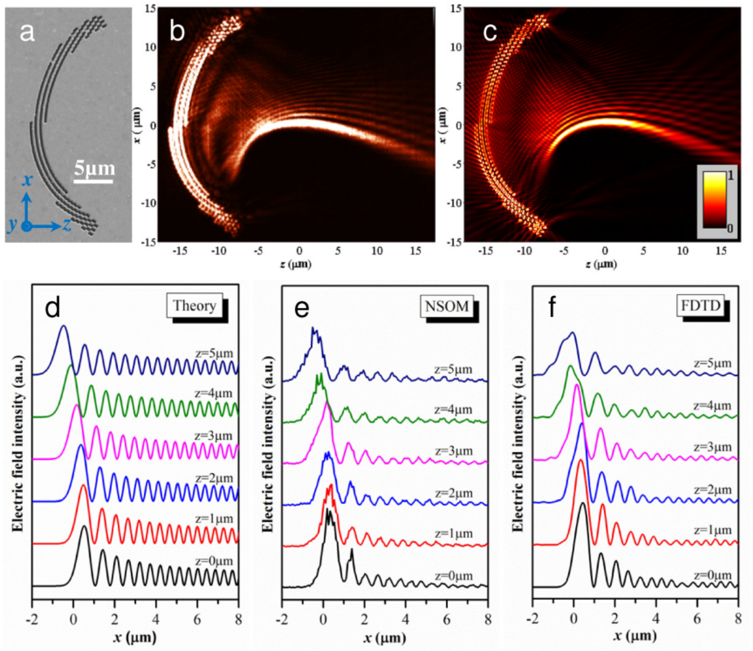

Figure. S7: (**a**) SEM micrograph of three-period nanostructures used to synthesize the PWB for $a = 60$. (**b**) NSOM measurement and (**c**) FDTD calculation results of electric field intensity distributions. The snapshots of the beam profiles at various propagation distances obtained from theory, NSOM and FDTD are shown in (**d**), (**e**) and (**f**) respectively.

References

1 Baker BB, Copson ET. *The Mathematical Theory of Huygens' Principle*. Oxford: Oxford University Press; 1950.

2 Abramowitz M, Stegun IA. *Handbook of mathematical functions*. New York: Dover Publications; 1972.

3 Bandres MA, Gutiérrez-Vega JC, Chávez-Cerda S. *Parabolic nondiffracting optical wave fields*. *Opt. Lett.* 2004; **29**: 44-46.

4 Morse PM, Feshbach H. *Methods of Theoretical Physics*. New York: McGraw-Hill; 1953.

5 Zhang P, Hu Y, Li TC, Cannan D, Yin X B, *et al*. *Nonparaxial Mathieu and Weber accelerating beams*. Phys. Rev. Lett. 2012; **109**: 193901.
